# Preventive effect of Agnucastoside C against Isoproterenol-induced myocardial injury

**DOI:** 10.1038/s41598-017-16075-0

**Published:** 2017-11-23

**Authors:** Sunanda Panda, Anand Kar, Sagarika Biswas

**Affiliations:** 1 School of Life Sciences, Takshashila Campus, Devi Ahilya University, Indore, India; 2 Department of Genomics & Molecular Medicine, CSIR-Institute of Genomics and Integrative Biology, New Delhi, India

## Abstract

An iridoid glycoside, agnucastoside C (ACC) was isolated from the leaves of *Moringa oliefera* and its cardio protective potential was investigated in adult rats by examining the effects of this test compound, ACC at 30 mg/kg for 14 days in isoproterenol (100 mg/kg)-induced myocardial injury. Isoproterenol (ISO) administration induced the myocardial injury as evidenced by the altered ECG pattern with ST-segment elevation and an increase in the levels of cardiac injury markers including troponin-I, creatine kinase-MB, alanine transaminase, aspartate transaminase, lactate dehydrogenase; inflammatory markers, interleukine-6 and tumor necrosis factor. In this group, there was also an increase in cardiac lipid peroxidation and a decrease in cellular antioxidants. However, pretreatment with ACC maintained the normal ECG pattern and nearly normal levels of all the cardiac markers in ISO-induced animals. Electron microscopic and histological studies also showed marked reduction in ISO-induced cardiac damages including infarct size by ACC. Analysis by 2-DE revealed the involvement of 19 different cardiac proteins, associated with energy metabolism, oxidative stress and maintenance of cytoskeleton. The expression of those proteins were altered by ISO, but maintained in ACC pretreated rats. Our findings reveal the potential of isolated ACC in the prevention of myocardial damage.

## Introduction

Coronary artery disease (CAD) is a worldwide health problem that very often leads to myocardial infarction (MI). In fact, MI, an acute condition of necrosis of myocardium is the most lethal manifestation of CAD that normally results when blood flow stops to a part of the heart, causing damage to the heart muscles^1^. This can be studied by examining the changes in different patho-physiological indices including electrocardiogram (ECG), serum levels of troponin-I, cardiac enzyme markers, and by imaging cardiac tissues^2^.

For the prevention & treatment of CAD several conventional medicines have been investigated earlier. These include beta-blockers and calcium antagonists. However, these have been found to be either not very effective or with side effects^3–5^. Very often alternative medicines, particularly plant based drugs are considered more effective and safe.

It is now well understood that oxidative stress, produced by isoproterenol (ISO) is mediated through free radicals or reactive oxygen species (ROS), as evidenced by marked increase in tissue lipid peroxidation (LPO) and decreased levels of antioxidants such as superoxide dismutase (SOD), catalase (CAT) and reduced glutathione (GSH), which play a major role in the protection from myocardial infarction (MI)^6^. Therefore, any compound known as strong antioxidant is believed to protect the cardiac tissues from MI. In fact, some experimental and clinical studies have shown that myocardial infarct size can be limited by increasing endogenous antioxidants and suppression of free radical generation^7^.

With this concept, it was suggested that use of some phytochemicals may protect an individual from CVD^8^. However, lack of detailed investigation on those isolated compounds has prevented them from their therapeutic use. Obviously, there is/was a need to search a suitable active compound from plants for regulating MI.

*Moringa oleifera* Lam. (Family, Moringaceae), commonly known as drumstick tree is a widely cultivated species in tropics and subtropics of Asia and Africa. It was also utilized by the ancient Roman, Greeks and Egyptians. India has the prime position in the cultivation and production of *M. oleifera* ^9^. It possesses many pharmacological characters including anti-inflammatory, antihypertensive, cholesterol lowering, antioxidative and hepatoprotective properties^10–12^. Our earlier studies also reported that active components of its leaves exhibit cardio protection^11,12^. As in some reports, iridoid glycosides such as Cornin, Picroside II and Catalpol were also shown to be effective in cardio protection^13–15^, in this investigation we thought of isolating an iridoid glycoside from the methanolic extract of its leaves and then to investigate its preventing effects on myocardial injury, if any. On the basis of its spectroscopic analyses, as described earlier in previous report^16^, the isolated compound was identified as agnucastoside-C (7-O-trans-p-coumaroyl-6-O-trans-caffeoyl-8-epiloganic acid). We primarily evaluated its effects against ISO-induced myocardial injury *in vivo*. To the best of our knowledge, the effects of isolated agnucastoside C (ACC) on ISO-induced myocardial damage in rats were not investigated till date by any one. As the molecule is associated with coumaryol and caffeoyl moieties which were reported to be cardioprotecive^17,18^, we speculated that the test agnucastoside C may have the potential to protect MI. To test our hypothesis, *in vivo* experiments were performed to evaluate the preventive effects of the test compound, if any, considering the changes in cardiac markers, lipid peroxidation, antioxidants, histopathological and ultra-structural changes as main indices. We also evaluated the possible antimyocardial ischemic mechanism of ACC through proteomic analyses.

Isoproterenol is a synthetic catecholamine and beta adrenergic agonist, which causes severe stress in the myocardium, resulting in infarct like necrosis of the heart muscle^19^. Therefore, experimental induction of MI by ISO in animals is a well-established phenomenon to study the potential of different cardio protective agents. ISO administration is followed by numerous pathophysiology and biochemical changes such as lipid peroxidation, hyperlipidemia, inflammation, myocyte loss, necrosis, increased calcium overload, alterations of membrane permeability etc. and is believed to be comparable to those taking place in human myocardial alterations^20^. We therefore isolated a compound from *Moringa oliefera* leaves and studied its cardio-protective and toxic effects, if any in ISO-induced rats.

For the evaluation of the preventive effects of the test compound, changes in cardiac markers, lipid peroxidation, antioxidants, histopathology and ultra-structure of heart were considered as main indices. The most extensively applied separation method in proteome analysis, two dimensional electrophoresis (2-DE) was also used for analyzing the changes of differential proteins of cardiac tissues to evaluate the possible anti-myocardial ischemic mechanism of ACC.

## Results

### Characterization of the isolated compound

The isolated compound was obtained as brown powder. Its molecular formula was determined as C_34_H_36_O_15_ by HRESIMS that exhibited the [M + Na]^+^ peak at m/z 707.224 (Fig. S1, Supplementary section). Its ^1^H and ^13^C NMR spectra indicated that this compound is an iridoid glycoside. In addition, UV (215, 277 nm) and IR (3428, 1690, 1630, 1510, and 1431) absorptions suggested the presence of hydroxyl, conjugated carboxylic enol–ether system and aromatic groups. The ^1^H and ^13^C NMR data of the compound are similar to those reported earlier for 8-epiloganic acid^16^ (For the data of NMR, Mass,UV and IR, please see the supplementary section). In ^13^C NMR data indicated the presence of 8-epiloganic acid, coumaroyl and caffeoyl units. In addition, the HRESIMS data indicated three main fragments and the ion fragments were, 365 [707–342(C_15_H_18_O_9_)]^+^, 432[707–275 (C_6_H_10_O_6_ + 2H_2_O + CO_2_H + O)]^+^ and 203[707–504 C_15_H_17_O_9_ + C_9_H_7_O_3_]^+^.

**Figure 1:**
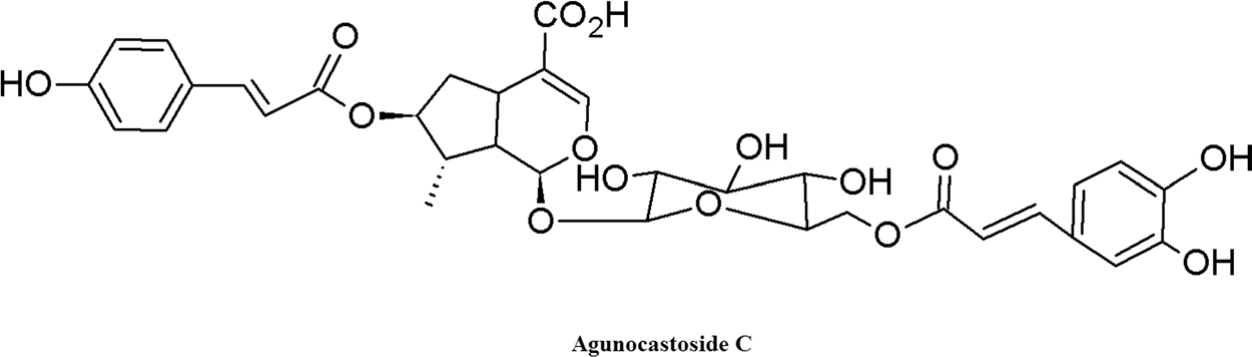
Structure of the isolated agnucastoside *C* (7-O-trans-p-coumaroyl-6-O-trans-caffeoyl-8-epiloganic acid).

On the basis of fragmentation pattern, a linkage of the caffeoyl unit to the hydroxy group at C-6 and of the coumaroyl unit to the hydroxy group of C-7 was established, which is similar to an earlier report^16^. Thus, the compound was elucidated to be 7-O-trans-p-coumaroyl-6-O-trans-caffeoyl-8-epiloganic acid (agnucastoside C) (Fig. 1).

### Changes in heart weight, body weight and electrocardiogram

Although at the end of the experiment, no significant alteration in body weight (b.wt.) of ISO-treated animals was observed (Table-S1), the heart weight and the ratio of heart weight to b.wt. were increased in this group, while rats pretreated with ACC alone or ACC + ISO exhibited a decrease in heart to b.wt. ratio when compared to that of ISO–induced animals. In ECG, significantly elevated ST segments were observed after ISO treatment, which were reduced in the ACC pretreated ISO-induced animals. Similarly, the heart rate was increased in ISO-treated animals, whereas pretreatment with ACC decreased the same [Supplementary section, Fig. S2(a) and (b)].

### ACC reduced serum cardiac markers and cytokine levels

**Figure 2:**
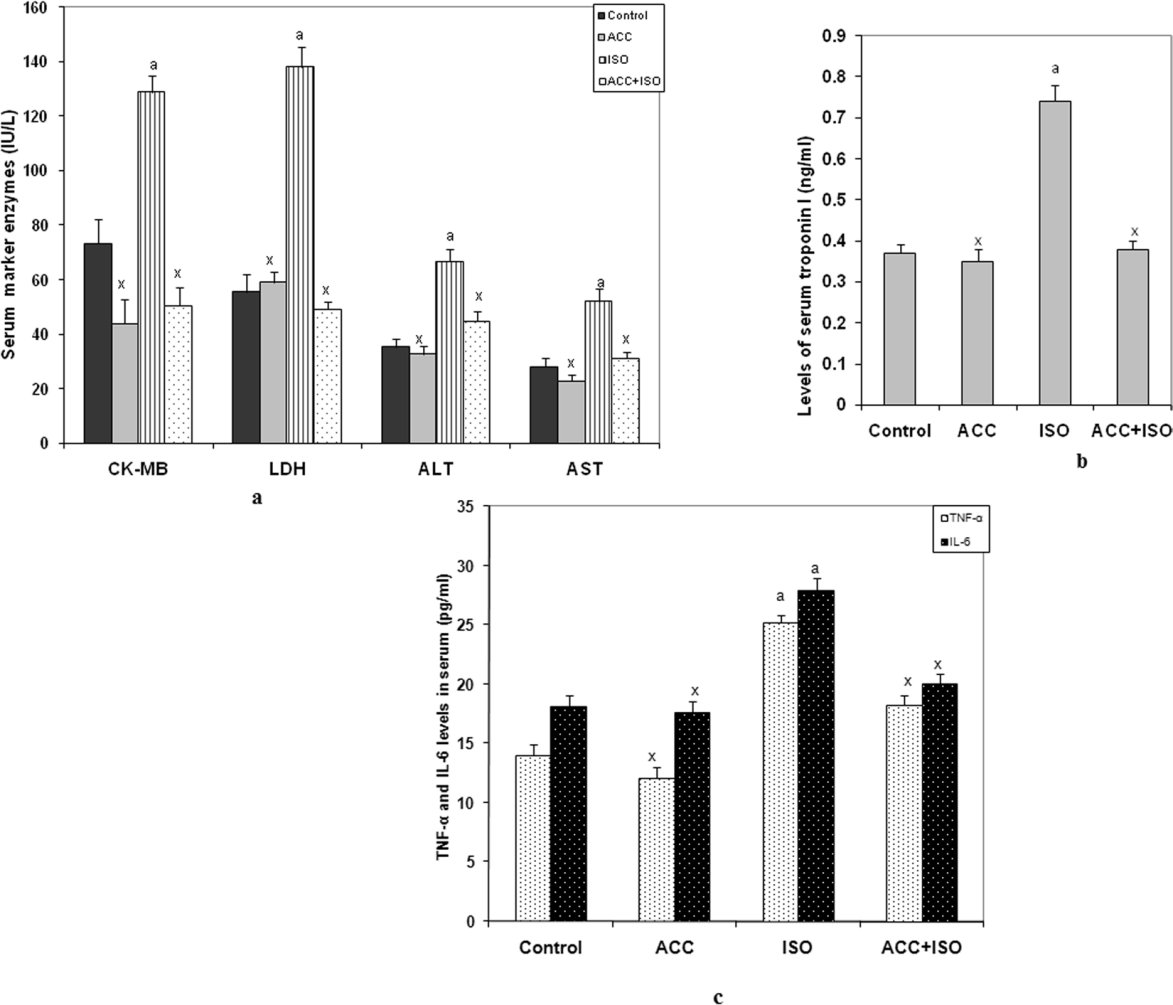
(**a**) Changes in serum creatine kinase–MB (CK–MB, in IU/L), lactate dehydrogenase (in LDH, IU/L), Alanine transaminase (ALT, in IU/L) and Aspartate transaminase (AST,in IU/L) following the administration of either ISO alone or with ACC + ISO. Each vertical bar represents the mean ± SEM (n = 7), analyzed by one way analysis of variance (ANOVA) followed by post-hoc comparisons by Student Newman-Keuls test. ^a^P < 0.001, as compared to the respective control value. ^x^P < 0.001 as compared to the respective value of the ISO-induced animals. (**b**) Changes in serum cardiac troponin I (cTnI, ng/ml). ACC pretreatment to ISO-induced animals decreased its level. Data are expressed as mean ± SEM; n = 7 and analyzed by one way analysis of variance (ANOVA) followed by post-hoc comparisons by Student Newman-Keuls test. ^a^P < 0.001, as compared to the respective control value. ^x^P < 0.001 as compared to the respective value of the ISO- induced animals. (**c**) Changes in serum TNF-α and IL-6 levels (pg/ml). ACC pretreatment to ISO-induced animals decreased TNF-α and IL-6 levels. Data are expressed as mean ± SEM; n = 7 and analyzed by one way analysis of variance (ANOVA) followed by post-hoc comparisons by Student Newman-Keuls test. ^a^P < 0.001, as compared to the respective control value. ^x^P < 0.001 as compared to the respective value of the ISO- induced animals.

While ISO-induced animals showed a significant increase in the activities of serum myocardial injury marker enzymes, creatine kinase (CK-MB), serum lactate dehydrogenase (LDH), alanine aminotransferase (ALT) and aspartate aminotransferase (AST) when compared with that of control rats, pretreatment with ACC maintained nearly normal values of all these serum diagnostic maker enzymes in ISO-induced animals (Fig. 2a). Rats induced with ISO also exhibited increased levels of cardiac troponin I (cTnI) in serum, but pretreatment with ACC to ISO-induced rats, significantly decreased its level as compared to ISO induced rats (Fig. 2b). In ISO-induced rats, serum levels of both tumor necrosis factor- α (TNF-α) and interleukin-6 (IL-6) were increased. However, ACC inhibited the overproduction of these two in ISO-induced animals and normalized the same (Fig. 2c).

### ACC pretreatment improved the lipid profile

ISO induced rats exhibited a significant rise in the levels of serum total cholesterol(TC), triglyceride (TG), low density lipoprotein cholesterol (LDL-C) and very low density lipoprotein cholesterol (VLDL-C) with a parallel decrease in high density lipoprotein cholesterol (HDL-C). However, pretreatment with ACC in ISO-induced animals restored all these serum lipids to normal levels (Table 1).

**Table 1:** Alterations in the levels of total cholesterol(TC), triglyceride (TG), low density lipoprotein cholesterol (LDL-C), very low density lipoprotein cholesterol (VLDL-C) and high density lipoprotein cholesterol (HDL-C) in normal (Ctrl), isoproterenol (ISO,100 mg/kg), (ACC, 30 mg/kg) alone and in ISO (100 mg/kg) + ACC (30 mg/kg) treated rats.

Groups	TG mg/dl	TC mg/dl	LDL-C mg/dl	VLDL-C mg/dl	HDL-C mg/dl
Control	98.73 ± 3.16	74.15 ± 2.88	22.04 ± 1.72	19.60 ± 0.597	36.06 ± 1.44
ACC	66.24^x^ ± 2.55	61.55^x^ ± 3.38	8.93^x^ ± 1.55	12.24^x^ ± 0.30	37.97^x^ ± 1.43
ISO	161.85^a^ ± 9.45	101.89^a^ ± 3.15	54.28^a^ ± 2.63	32.64^a^ ± 2.39	17.81^a^ ± 1.60
ISO + ACC	84.65^x^ ± 3.92	68.72^x^ ± 3.99	20.26^x^ ± 2.37	16.88^x^ ± 0.54	33.76^x^ ± 2.43

Data are means ± S.E.M. (n = 7). ^a^ *P* < 0.001 as compared to the respective control values. ^x^ *P* < 0.001 as compared to the respective values of the isoproterenol induced animals. (one-way ANOVA).

### ACC pretreatment inhibited LPO and increased antioxidant levels

**Figure 3:**
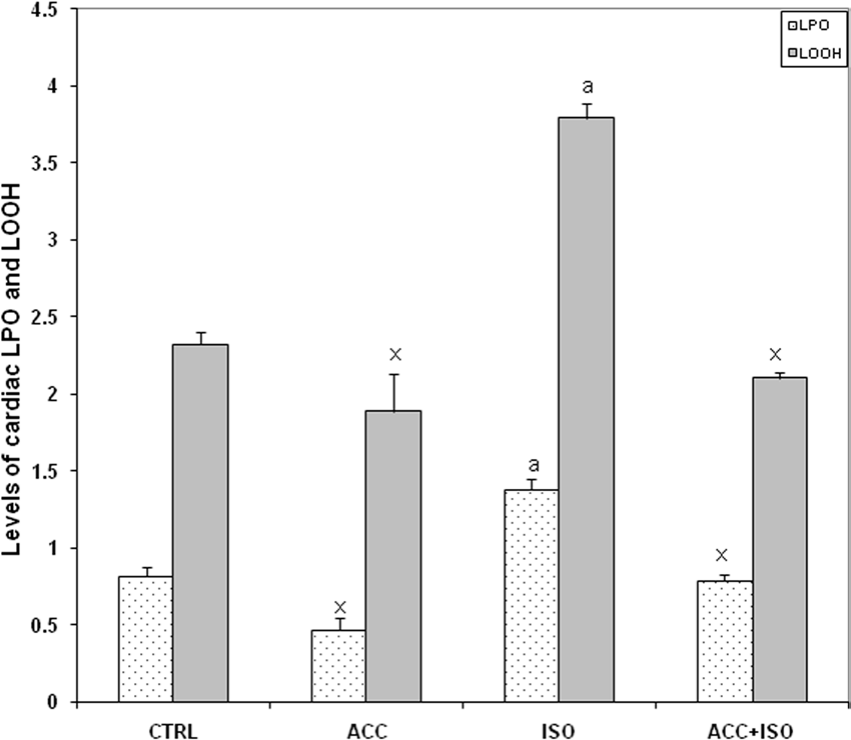
Levels of thiobarbituric acid reactive substances (TBARS, nM MDA formed/h/mg protein) and lipid hydroperoxides (LOOH, nM/mg protein) in cardiac tissues following the administration of either ISO alone or with ACC. Each vertical bar represents the mean ± SEM (n = 7) analyzed by one way analysis of variance (ANOVA) followed by post-hoc comparisons by Student Newman-Keuls test. ^a^P < 0.001 as compared to the respective control value. ^x^P < 0.001 as compared to the respective value of the ISO- induced animals.

In rats treated with ISO, a significant increase in the levels of thiobarbituric reactive substances (TBARS) and lipid hydroperoxides (LOOH) was observed, while there was a significant decrease in the activities of enzyme antioxidants such as superoxide dismutase (SOD), catalase (CAT), glutathione peroxidase (GPx) and in the levels of reduced glutathione (GSH) in the heart tissues. However, pretreatment with ACC decreased considerably the levels of TBARS and LOOH and increased the activities /levels of these antioxidants as compared to that of respective ISO control values (Fig. 3 and Table 2).

**Table 2:** Alterations in enzymatic and non-enzymatic anti-oxidants following ISO (100 mg/ kg), ISO + ACC (30 mg/ kg) and ACC (30 mg/kg) in the cardiac tissues of rats

Group(s)	SOD	CAT μ (moles of H_2_O_2_ decomposed /min/mg protein)	GSH (μmoles GSH/mg Protein	GPx (μmoles of GSH oxidized/mg protein)
Control	6.97 ± 0.52	45.27 ± 6.10	7.99 ± 0.37	8.27 ± 0.98
ACC	7.08^x^ ± 0.59	42.95^y^ ± 5.51	8.25^x^ ± 0.62	8.89^x^ ± 0.79
ISO	3.28^a^ ± 0.21	23.45^a^ ± 4.09	3.09^a^ ± 0.47	3.94^a^ ± 0.86
ACC + ISO	5.82^x^ ± 0.39	51.05^y^ ± 4.98	7.12^x^ ± 0.34	6.19^y^ ± 0.85

Data are means ± SEM, n = 7. Significance was determined by One-Way ANOVA followed by Student Newman-Keuls Post-hoc test: ^a^P < 0.001, as compared to the respective control values. ^x^ *P* 0.001 and ^y^ *P* < 0.01, as compared to the respective values of the ISO- induced animals.

### Proteomic analyses identified 19 different proteins

**Figure 4:**
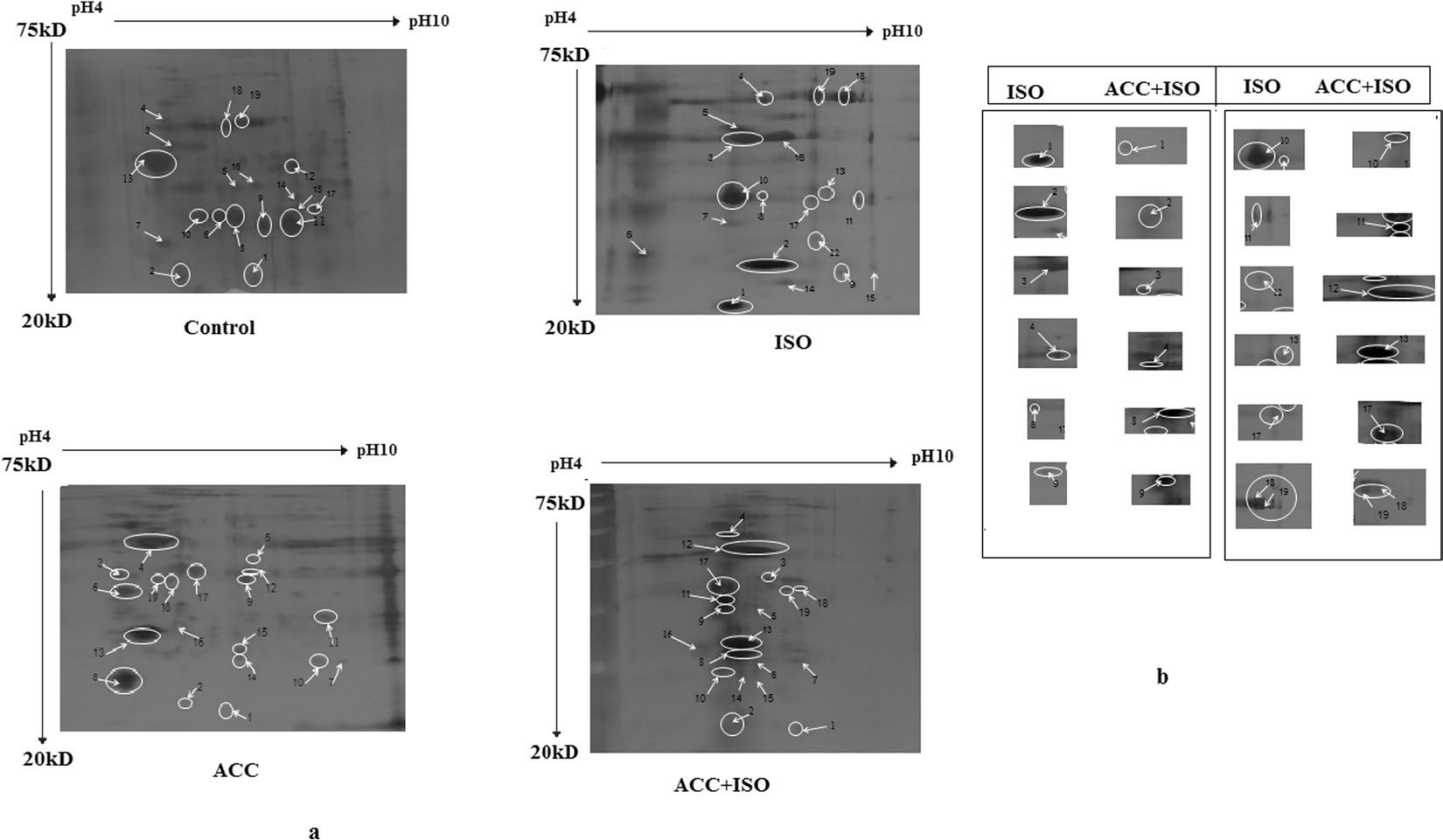
(**a**) Representative 2D gel images, obtained from heart protein extracts in control, ACC, ISO and ACC pretreated ISO-induced rats. Differential proteins are marked by arrow and number. (**b**) Close up area of the gels showing variation in intensity of the differentially expressed protein spots in ACC + ISO and ISO-induced rats.

In the present study, cardiac proteins, separated by 2-DE, resulted in 45 differentially expressed protein spots and 19 amongst them were identified properly (Fig. 4a). Relative spot intensity of the proteins in ISO and ISO + ACC groups (calculated by taking control group as standard) and the complete list of proteins identified by MS are listed in Table 3. The proteins associated with energy metabolism, stress/heat shock and cytoskeletal function indicated their increased expression. Proteins associated with glycolysis and phosphocreatine, LDH-B and CK in ACC-pretreated ISO-induced animals were also over expressed; whereas myosin light polypeptide 3, α-cardiac actin and the cytoskeleton protein were down regulated. Moreover, stress-induced/heat shock proteins such as glucose-regulated protein, Grp58, αB-crystallin and Hsp27, all were down-regulated in ACC pretreated ISO-induced group.

**Table 3:** Identified altered proteins in ISO-induced agnucastoside *C* pretreated with respect to ISO-treated group.

Spot No.	Protein identity	Function	NCBI No.	Relative spot Intensity (%) (Control, 100)^b^		Experimental values theoretical values (pI /kDa)	Matched sequence
				ISO	ACC + ISO		
1.	^a^Heat shock protein	heat shock protein	14010865	174^##^ ± 14	67** ± 4.7	5.7/22 (6.1/23)	3
2.	^a^alpha B-crystallin	heat shock protein	57580	770^##^ ± 34.2	72** ± 3.4	6.5/21 (6.8/19)	3
3.	^a^Glucose regulated protein, 58 kDa	stress-induced	38382858	67^##^ ± 3.9	24** ± 1.7	6.0/57 (5.9/57)	3
4.	Glucose regulated protein 78 kDa	stress-induced	25742763	52^##^ ± 4.5	159** ± 6.1	6.0/57 (5.07/71)	3
5.	NADH dehydrogenase 1 subunit alpha 10	Rsp-Chain	32996721	107## ± 14	39** ± 12	6.3/41 (7.1/40)	6
6.	NADH ubiquinone oxidoreductase (24-kDa subunit)	Rsp-Chain	83305118	58^#^ ± 6.4	99 ** ± 4.4	4.8/22 (6.0/26)	2
7.	ATP synthase, H( + )-transporting	Rsp-Chain	57029	123# ± 6.9	76** ± 7.6	6.2/27 (7.0/26)	9
8.	^a^Lactate Dehydrogenase-B	Glycolysis	6981146	28^##^ ± 1.6	124** ± 11.7	6.6/0.34 (5.7/37)	3
9.	Malate dehydrogenase 1	TCA-cycle	15100179	37^#^ ± 6.5	102* ± 7.9	6.3/36 (6.2/36)	10
10.	Triose phosphate isomerase	Glycolysis	38512111	201^##^ ± 10	95** ± 6.5	7.0/22 (7.0/27)	3
11.	Aldolase A	Glycolysis	202837	121^##^ ± 14	174** ± 15	7.6/37 (8.3/39)	2
12.	^a^Long-chain acyl-CoA Dehydrogenase	β-oxidation	6978431 26	26^##^ ± 2.2	105** ± 12.2	6.97/48 (7.6/48)	3
13	^a^Creatine Kinase	Phosphocreatine	6978661	68^##^ ± 4.9	275** ± 15.9	6.0/43 (6.5/43)	7
14	^a^Myosin light chain Polypeptide3	cytoskeleton	6981240	48^##^ ± 3.1	26* ± 4.3	4.8/23(5.0/22)	4
15.	^a^Myosin heavy chain Polypeptide6	cytoskeleton	6981240	32^##^ ± 3.4	21* ± 1.9	4.8/23(5.0/22)	5
16.	^a^α-actin	cytoskeleton	695390	116^##^ ± 16.8	42** ± 5.4	5.4/44 (5.2/42)	2
17.	^a^Apolipoprotein A1	Other protein	146345369	35^##^ ± 3.1	101** ± 9.2	5.6/35 (5.5/30)	4
18.	CaMKIIδ_B_ isoform	calcium-binding proteins	535179	84^##^ ± 2.5	15** ± 3.1	6.1/52(5.6/54)	20
19.	CaMKIIδ_C_ isoform	calcium-binding proteins	545960	60^##^ ± 3.6	24^*^ ± 2.2	6.1/52 (6.2/52)	17

^a^Theoretical molecular mass and pI were derived from the amino acid sequences in NCBI.

*P < 0.05, **P < 0.001 compared to the value of control group; ^#^ *P* < 0.05, ^##^ *P* < 0.001, compared to the value of the ISO treated group.

Selected regions of 2D image showing examples of protein spots with significantly altered expression in ISO and ACC + ISO groups are shown in Fig. 4(b) and information on each numbered spot is listed in Table 3.

### Comparative proteome profiling and the expression of 12 prominent proteins

The analyses of the protein expression profiles of heart tissues of rats from ISO and ACC + ISO treated rats resulted in the identification of several differentially expressed protein spots. These proteins are shown in Fig. 4(b) and information on each numbered spot is listed in Table 3. They include, heat shock protein, alpha B-crystallin, glucose regulated-58kDa stress protein, glucose regulated-78kDa stress protein, lactate dehydrogenase, triose phosphate isomerase, aldolase A, long chain acyl-Co A dehydrogenase, creatine kinase, apolipo protein A1, CaMKII_δB_ and CaMKII_δC_ calcium binding proteins (spot no. 1, 2, 3, 4, 8, 10, 11, 12, 13, 17, 18, 19 respectively).

### ACC protected ISO-induced myocardial tissue necrosis

Images of triphenyl tetrazolium chloride (TTC) stained ventricular slices cut from four different hearts indicated prominent whitish TTC-negative areas in the ISO-treated hearts, indicating the presence of infarction. The infracted areas were patchy and more diffusely located on the myocardium and did not stain with TTC.

**Figure 5:**
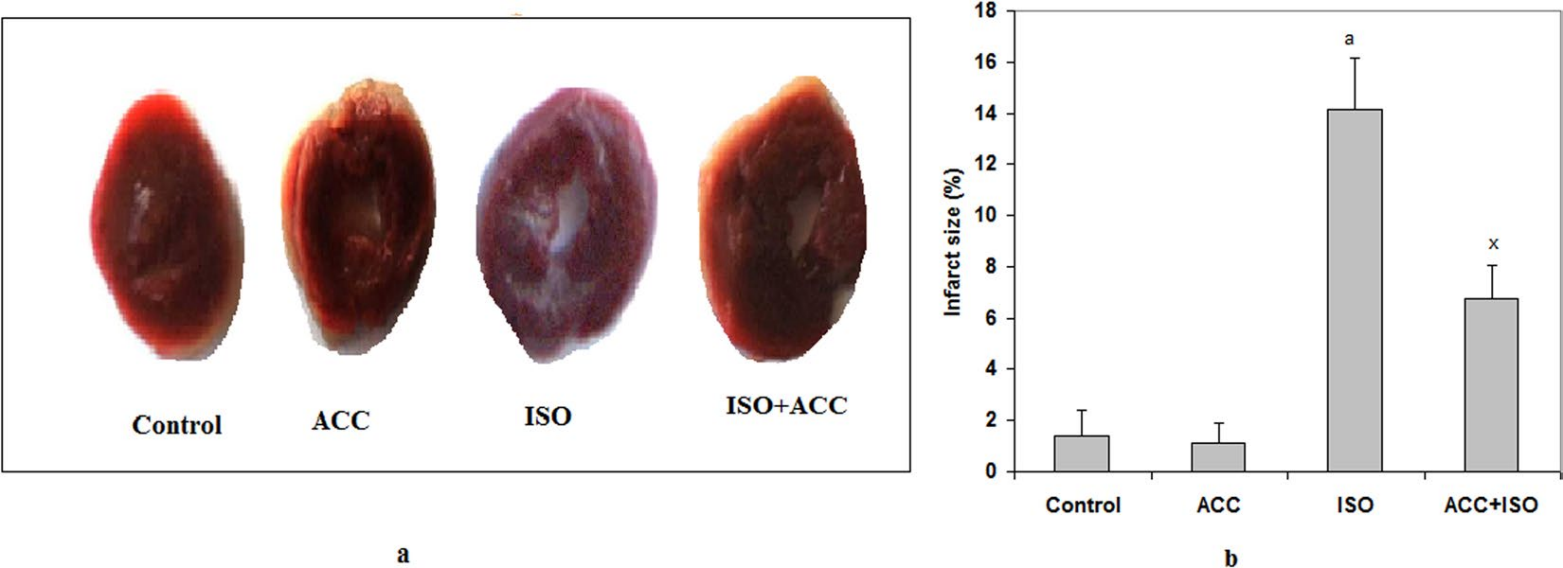
(**a**) TTC staining assay in rat heart tissue. Image of normal control heart showing the completely viable myocardial tissues, Image of ISO-induced infracted tissue showing more necrotic areas which do not stain with TTC, heart pretreated with ACC + ISO reduced necrosis and showed positively stained viable heart tissue. (**b**) Effects of ACC pretreatment on infarct size in ISO-induced MI. The infarct size is expressed as % area to the total ventricular area. Each vertical bar represents the mean ± SEM (n = 7), analyzed by one way analysis of variance (ANOVA) followed by post-hoc comparisons by Student Newman-Keuls test. ^a^P < 0.001, as compared to the respective control value. ^x^P < 0.001 as compared to the respective value of the ISO-induced animals.

In contrast to the effects of ISO, the control and ACC-only treated hearts appeared mostly red (dark, TTC positive), suggesting tissue viability (Fig. 5a). The quantification of infarct size in whole ventricles is summarized in Fig. 5(b) showing ISO induced increases in infarct size and prevention of the ISO-induced infarction and by pre-treatment with ACC. Treatment with ACC alone did not indicate injury to the myocardium.

The area of necrosis as determined by TTC staining, indicated that the heart section from normal control rats and ACC pretreated ISO-induced group had a major portion, stained positively, showing tissue viability and the presence of LDH. ISO administered rat exhibited extensive necrosis, clearly visible as pale gray or colorless area.

### ACC alleviated ISO-induced histological and ultra structural changes in the myocardial tissues

**Figure 6:**
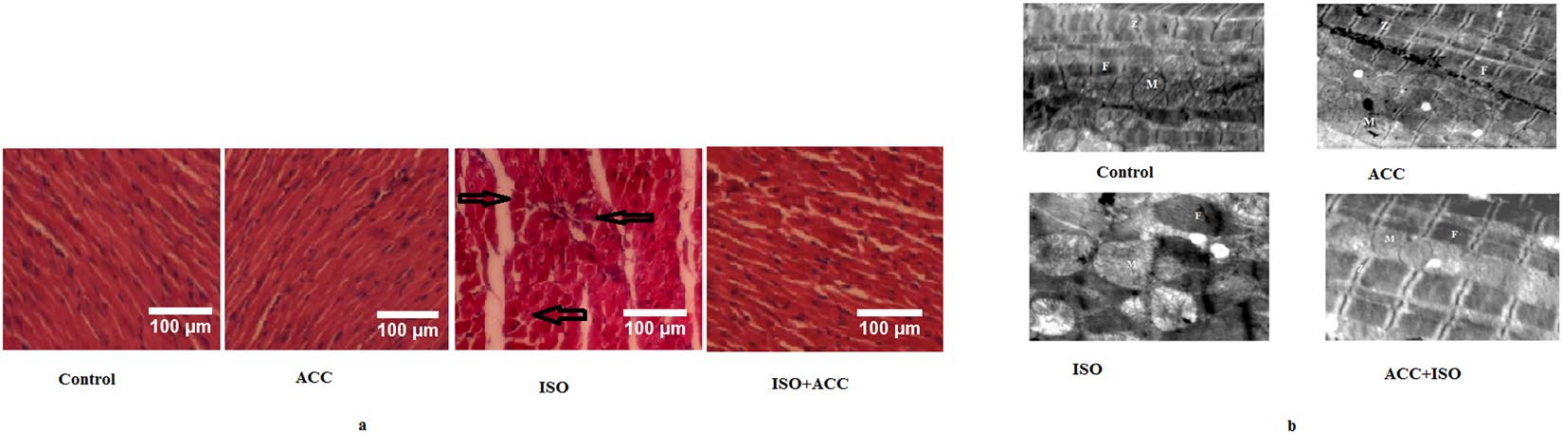
(**a**) Histopathological changes in heart of Control, ISO, AAC + ISO and only AAC administered rats. [H & E (x200)]. Sections of heart from control group showing normal architecture. ISO-induced rats showing marked infiltration of inflammatory cells, necrosis and disruption of cardiac myofibres. Rat heart of pretreated AAC revealed normal architecture of heart. Arrow indicates cellular infiltrations of inflammatory cells. (**b**) Electron micrograph of a heart of a control rat showing regular arrangement of the myofibrils (F), mitochondria (M) and regular Z lines. Electron micrograph of a heart of a ISO-induced rat showing disruption of myocardial fibers with swelling of heart mitochondria, irregular shape and size. Electron micrograph of a heart of AAC treated rat showing nearly regular arrangement of the myofibrils, normal mitochondria and regular Z lines.

Administration of ISO caused cellular necrosis, interstitial edema, vacuolization of myofibrils, and inflammatory cell infiltration in cardiac tissues as well as massive disruption and fragmentation of the myofibers. However, rats, pretreated with test ACC in ISO-induced animals nearly normalized the cellular architecture by decreasing necrosis, and reducing infiltration of inflammatory cells (Fig. 6a).

Transmission electron microscopy (TEM) analyses of ISO–induced myocardial tissues revealed fragmented myofibrils, swelling of mitochondria with changes in shape and size and discontinuity of Z lines; while EM study of control, agnucastoside and agnucastoside + ISO heart showed regular Z lines and arrangement of the myofibrils along with a normal appearance of mitochondria (Fig. 6b).

## Materials and Methods

### Plant Material

Leaves of *M. oleifera Lam*. (family, Moringaceae) were collected from our botanical garden and authenticated by the botanist, Prof. A. Jajoo, D.A. University, Indore, India. A voucher specimen (MO/05–99) has been deposited in departmental herbarium for future reference.

### Chemicals

Isoproterenol hydrochloride and protease inhibitor cocktail were purchased from Sigma Chemical Co., St. Louis, MO, USA; while the chemicals used for biochemical assays were products of E. Merck Ltd., Mumbai, India. All other chemicals and reagents used were procured from Hi-Media, Mumbai, India. Thio-barbituric acid (TBA), sodium dodecyl sulphate (SDS), Ellman’s reagent and m-phosphoric acid were obtained from E. Merck Ltd., Mumbai, India. While assay kits for CK-MB, LDH, AST and ALT were purchased from Teco Diagnostic,USA; for total cholesterol, Triglyceride and HDL-C, kits were obtained from Span diagnostics Pvt. Ltd., Surat, India. The TNF-α and IL-6 specific ELISA kits were from Ray Biotech, Inc., USA and cardiac troponin-I (cTnI) kit was procured from the Ortho-Clinical Diagnostics, Inc. New York, USA. While Bisacrylamide, Tris hydroxymethyl aminomethane (Tris), glycine, N,N, NU,NU-tetramethylethyldiamide, ammonium persulfate, glycerol, ultra pure urea, 2D cleanup kit and 2D Quant kit were purchased from GE Healthcare, Amersham, Freiburg, Germany; Acrylamide, dithiothreitol, 3–3–1-propane-sulfonate, agarose and Iodoacetamide were purchased from Fluka BioChemika, Buchs, Switzerland.

### Animals

Adult male albino Wistar rats (9 week old), each weighing 180 ± 10 g were housed in polypropylene cages in a standard light/dark (14 h light:10 h dark) cycle and maintained in a temperature (27 ± 1 °C) controlled room with the provision of laboratory feed (Gold Mohur feed, Hindustan Lever Limited, Mumbai, India) and water *ad libitum*. Animals were acclimated to their surrounding for 7 days before experimentation.

### Animal ethics

Guidelines of our Institutional Animal Ethical Committee (IAEC), registered with the Ministry of Social Justice and Empowerment, Government of India (registration No. is 779/Po/Ere/S/03/ CPCSEA) were adhered to. The departmental ethical committee (School of Life Science, DAVV, Indore, India) had also approved the experimental procedure including the maintenance/handing of animals and administration of drugs.

## Method

### Extraction and Isolation of active compound

Shade dried leaf powder (300 g) of *Moringa* *olifera* was used in the study. The defatted aqueous acetone (70%) extract (135 g) was suspended in distilled water (DW) and after filtering the insoluble materials; the filtrate was chromatographed over a sephadex LH-20 column using MeOH (10 to 100%). In brief, the dried MeOH extract (40 g) was fractionated by solid-phase-extraction (SPE) on ODS-5 octadecyl cartridge using a step gradient of MeOH-water mixture (10:90, 20:80, 40:60, 60:40, 80:20 and 100:0). All fractions were dried using a rotary evaporator at 40 °C. The 100% SPE fraction (yield, 390 mg, i.e, 1.3 g/Kg) showed positive result in Wieffering field test indicating the presence of an iridoid. The isolated iridoid glycoside was subjected to spectroscopic analyses (details in supplementary section) and by comparing the available values in the literature, as described earlier^16^, it was identified as 7-O-trans-p-coumaroyl-6-O-trans-caffeoyl-8-epiloganic acid.

### Pilot study for dose fixation of test ACC

To find out the optimum dose of test ACC, a preliminary study was conducted with four different doses of isolated agunocastoside (15, 30, 60 and 90 mg/kg; p.o.) in ISO treated rats. For this, experimental animals were divided into six groups of seven rats each. The dose screening was determined by evaluating the changes in ECG (ST-segment), serum CK-MB and cardiac LPO. It was observed that after 14 days of test agnucastoside pretreatment, out of four doses, 30 mg/kg significantly decreased the serum CK–MB activity and cardiac LPO and showed normal pattern of ST-segment in ECG indicating maximum cardio protective effects (data not shown). Hence this dose (30 mg/kg) was selected for further investigation.

### Detailed investigation on the protective effects of ACC

Twenty eight healthy male albino Wistar rats (9 weeks old and weighing 180 ± 10 g) were randomly divided into four groups (Group1, control; Group 2, ISO treated; Group 3, ACC treated and Group 4, ACC + ISO treated), with seven animals in each group. While animals of group I received 0.1 ml of vehicle (d.w., p.o.) and served as control; animals of group II also received 0.1 ml of d.w. for 14 days and then injected with ISO (s.c.,100 mg/kg) for 2 days (on 15th and 16th day) to induce myocardial injury^20^ and group III rats were administered with 30 mg/kg (p.o.) of test ACC (dissolved in d.w.) for 14 days. Animals of group IV were also treated with 30 mg/kg (p.o.) of test ACC for 14 days and then injected with ISO for next 2 days as done in group 2. At the end of the experiment (on 17th day), ECG recordings of the animals were taken. Overnight-fasted animals were killed by cervical decapitation, blood from each animal was collected and serum was separated for the evaluation of the cardiac markers and different lipids. Heart of each animal was removed quickly, washed and homogenized with phosphate-buffered saline (PBS, pH 7.4). The homogenates were centrifuged at 17,000 g for 30 min at 4 °C and the supernatant was used for biochemical estimations.

### Electrocardiographic analysis

On the last day of experiment, rats were anesthetized with ketamine (100 mg/kg, *i.m*.) and xylazine (10 mg/kg, i.m.)^13^, needle electrodes were inserted into paw pads, and connected to Cardiart 1081 (BPL) ECG machine. ECG recordings were made using 20 mm/mV sensitivity with a paper speed of 50 mm/s in lead II position and ST-segment elevation (expressed in mV) in normal and experimental animals were recorded.

### Determination of TNF-α, IL-6, cTnI,CK-MB, LDH, AST and ALT

The levels of TNF-α and IL-6 in serum were estimated using specific ELISA kits (Ray Biotech, Inc., USA). Cardiac troponin I was estimated in the serum also by the standard diagnostic kit (Vitros Immunodiagnostic Products), purchased from Ortho Clinical diagnostics, New York, USA.

Serum levels of CK-MB, LDH, ALT and AST were measured according to the manufacturer’s instruction using the standard estimation kits from Teco Diagnostics, CA, USA.

### Estimations of different lipids

Levels of serum TC, TG and HDL-C were estimated using the assay kits and LDL-C and very low density lipoprotein-cholesterol (VLDL-C) were calculated using the formula of Friedwald *et al*.^21^ as mentioned below.

$$\begin{array}{c}{\rm{V}}{\rm{L}}{\rm{D}}{\rm{L}}-C={\rm{T}}{\rm{r}}{\rm{i}}{\rm{g}}{\rm{l}}{\rm{y}}{\rm{c}}{\rm{e}}{\rm{r}}{\rm{i}}{\rm{d}}{\rm{e}}{\rm{s}}/5.\\ {\rm{L}}{\rm{D}}{\rm{L}}-C={\rm{T}}{\rm{o}}{\rm{t}}{\rm{a}}{\rm{l}}{\rm{c}}{\rm{h}}{\rm{o}}{\rm{l}}{\rm{e}}{\rm{s}}{\rm{t}}{\rm{e}}{\rm{r}}{\rm{o}}{\rm{l}}-({\rm{H}}{\rm{D}}{\rm{L}}-C+{\rm{V}}{\rm{L}}{\rm{D}}{\rm{L}}-C).\end{array}$$

The results were expressed as mg/dl.

### Estimations of myocardial necrosis and infarct size determination

These were performed by direct TTC assay as done earlier by us^22^. In brief, 2 mm thick ventricular transverse slices of the frozen heart were cut from apex to base, incubated in 1% TTC solution prepared in phosphate buffer (pH 7.4), kept for 30 min at 37 °C and then fixed with 10% formalin, then stored in the dark at room temperature for 24 hours. Finally, the photographs of heart slices were taken in camera with macro-lens. Infarct size was assessed by cumulative planimetery method. Images were analyzed manually using image processing soft ware (ImageJ, Version 1.44p, NIH, USA). The sum of infracted area of the individual slice was measured and divided by the total area of that slice to obtain the fraction of the slice. Results from individual slices from each heart were averaged on the basis of the weight to calculate the total ventricular ischemia for each heart^23^.

### Assessment of cardiac proteins

Protein extraction and 2-DE analysis were done following the method of Wang *et al*.^24^. Heart samples of four animals in each group were analyzed individually. Proteins (100 µg) were applied to a 17-cm immobilized pH gradient (IPG)-strip with a linear pH range of 4.0–10. Isoelectric focusing (IEF) using a multistep protocol (14 h for rehydration, 3 h at 500 V, 2.5 h at 4500 V, and finally at 4000 V). Sodium dodecyl sulfate polyacrylamide electrophoresis on 12% gels was used for the second dimension, following the silver staining. Briefly, gels were fixed in 50% (v/v) methanol and 12% (v/v) acetic acid for 2 h. Fixation was followed by two washing steps in 50% and 30% (v/v) ethanol. Gels were sensitized with 0.8 mM Na_2_S_2_O_3_ for 60 sec followed by three washes in water for 20 sec each. After silver impregnation with 0.2% (w/v) AgNO_3_ and 0.026% (v/v) formaldehyde for 20 min, again three water washes were given for 20 sec each. Development was done with 6% (w/v Na_2_S_2_O_3_, 0.0185% (v/v) formaldehyde and 16 mm Na_2_S_2_O_3_ and was stopped by 50% (v/v) methanol and 12% (v/v) acetic acid.

For spot detection, gel matching, and statistical analyses, PD Quest 7.1 software (Bio-Rad, Munich, Germany) was used. The protein spots were identified using the criteria, (1) at least twofold increase or decrease of spot intensity and passed through a paired Student ‘t’ test with a *P* value of at least <0.05, (2) sufficient spot resolution on the 2-DE gel allowing the exact excision of the spot for in-gel digest, and (3) the amount of protein available from the 2-DE gels being sufficient for matrix-assisted laser desorption-ionization time-of-flight mass spectrometry (MALDI-TOF) or electrospray ionization tandem mass spectrometry (ESI-MS/MS) analysis.

### In-gel digestion and mass spectrometry study

The protein of interest was cut, distained, and dehydrated. The dehydrated gel was then incubated in trypsin solution (0.1 mg/mL in 25 mM ammonium bicarbonate) for 20 h at 37 °C. The peptides were eluted in 0.7 mL matrix solution (a-Cyano-4-hydroxycinnamic acid in acetonitrile/water, acidified with 0.1% trifluoroacetic acid). The mixture was immediately spotted on the MALDI target and allowed to dry and crystallize and subjected to MALDI-TOF analysis for peptide mass finger printing and protein sequencing by using matrix assisted laser desorption ionization mass spectrometery (MALDI-TOF MS /MS) (Applied Biosystems, Life Technologies, USA).

MASCOT program against *Rattus norvegicus* species was done on the National Center for Biotechnology Information protein database (http://www.matrixscience.com). A maximum of one missed tryptic cleavage per peptide was allowed. Mass tolerance of 0.1 Da, and MS/MS tolerance of 0.1 Da were used, and variable modifications, such as carbamido-methylation for cysteine and oxidation for methionine were taken into account. Proteins were identified and classified only when there were at least two corresponding peptides and a significant database score. Those proteins were considered in which the calculated molecular weight and pI were similar to that of observed molecular weight and pI in the 2-DE.

### Comparative proteome profiling

In an effort to gain insight into the molecular mechanisms underlying the cardioprotective efficacy of ACC, we also performed a comparative analysis of the protein expression profiles of the heart tissues of ISO and ISO + ACC treated rats by image analysis of 2D gels using ‘progenesis same spots’ software, for the identification of differentially expressed protein spots.

### Histopathological examinations

After excising out the heart, the ventricular mass was sectioned from the apex to the base of heart and washed immediately with ice-cold normal saline and fixed in 4% buffered formalin and was embedded in paraffin and sectioned at 5 µm thickness and were stained with hematoxylin-eosin (H&E). The sections were examined under light microscope (Leica DFC 320 fluorescent microscope, type DM 5000B, Leica Microsystems Ltd) by the experienced pathologists who were blinded to the experimental protocol. Photomicrographs were taken at ×200 magnification.

The histopathological examination were scored and graded on the basis of severity of changes (presented in Table S2). Histological findings were classified into the system such as: (−) no changes; (+) mild (focal myocytes damage with slight degree of inflammatory process); (++) moderate (extensive myofibrillar degeneration and/or diffuse inflammatory process) and (+++) marked (massive areas of inflammation, edema, and necrosis^25^.

### Transmission electron microscopic study

This was performed by the method as described earlier by us^22^. Small pieces of heart were taken and rinsed in 0.1 M phosphate buffer (pH, 7.2). Approximately, 1-mm heart pieces were trimmed and immediately fixed into 2.5% ice cold glutaraldehyde in 0.1 M phosphate buffer (pH, 7.2) and kept at 4 °C for 12 h and post fixed with 1% buffered osmium tetroxide. Then, tissue processing for TEM study was carried out. The grids containing sections were stained with 2% uranyl acetate and 0.2% lead acetate. Then, the heart sections were examined under a transmission electron microscope (20,000x).

### Assessment of lipid peroxidation and antioxidants

Thiobarbituric reactive substances were estimated by the method described by Okhawa *et al*.^26^. Briefly, to 0.2 ml of tissue homogenate, 0.2 ml of 8.1% SDS, 1.5 ml of 20% acetic acid and 1.5 ml of 0.8% TBA were added in succession. Volume was made up to 4 ml with double distilled water. The mixture was incubated for 60 min at 95 °C in a temperature controlled water bath. After cooling, the pink colored complex was extracted with 5 ml of butanol:pyridine (15:1) mixture. Organic layer was separated and absorbance was observed at 532 nm. TBARS levels were determined from the standard curve of TBA adduct formation when various concentrations of commercially available 1, 1, 3, 3-tetraethoxypropane were subjected to the above procedure. Data are expressed as nM MDA formed/h/mg protein.

#### Lipid hydroperoxides estimation

Estimation of tissue lipid hydroperoxides was done by the method of Jiang *et al*.^27^.

Fox reagent (1.8 ml) was mixed with 0.2 ml of the cardiac tissue supernatant and incubated for 30 min at room temperature. The color developed was read at 560 nm.

#### Catalase estimation

Catalase was estimated following the method described by Aebi^28^. Hearts were homogenized at 4 °C (1:10) in 50 mM potassium phosphate buffer (pH 7.4) and centrifuged at 3000 g for 10 min. Supernatant (50 µl) was added to a 3.0-ml cuvette that contained 1.95 ml of 50 mM phosphate buffer (pH 7.0). Then 1.0 mL of 30 mM H_2_O_2_ was added and changes in absorbance were recorded for 30 s at 240 nm at an interval of 15 s. Catalase levels were determined by the standard curve obtained using known concentration of commercially available catalase. Finally level of catalase was expressed in μ moles of H_2_O_2_ decomposed/min/mg protein.

#### Superoxide dismutase estimation

Activity of SOD is determined following the pyrogallol auto-oxidation inhibition assay described by Marklund and Marklund^29^ that was based on the rate of auto-oxidation and is calculated from the increase in absorbance at 420 nm. In fact, this assay is based on the ability of SOD to scavenge superoxide anion radical, which decreases the overall rate of pyrogallol autoxidation. In brief, 200 μl of supernatant was diluted in phosphate buffer to make final volume of 2.0 ml. To this 0.8 ml tris -HCl buffer (50 mM Tris, 1 mM diethylene triamine pentACCetic acid, pH 8.2) and 0.2 ml of 2 mM pyrogallol were added. The reaction was initiated by the addition of 1 ml (0.2 mmol/l) pyrogallol and the increase in absorbance was recorded at 420 nm for 3 min in a UV/VIS spectrophotometer. A blank was prepared without addition of pyrogallol in the reaction system. One unit of enzyme activity is equivalent to 50% inhibition of the rate of auto oxidation of pyrogallol as determined by changes in absorbance at 420 nm, at 1 min intervals for 3 min. The enzyme activity was expressed as units/mg of tissue protein.

#### Estimation of reduced glutathione

Reduced glutathione in the heart tissues supernatant was estimated by the method of Ellman^30^. Protein free supernatant was obtained by addition of equal volume of 5% TCA to the tissue homogenate and centrifuged at 5000 rpm for 10 min. A total of 1.0 ml of supernatant was taken after centrifugation and 0.5 ml of Ellman’s reagent and 3.0 ml of 0.3 M disodium hydrogen phosphate were added. The yellow color developed was read at 412 nm. Total protein was assayed by the method of Lowry *et al*. 3^31^.

#### Estimation of glutathione peroxidase

The activity of GPx in the cardiac tissues was studied by the method of Rotruck *et al*.^32^. To 0.2 ml of tris buffer, 0.2 ml of ethylene diamine tetra accetic acid (EDTA), 0.1 ml of sodium azide, and 0.5 ml of tissue homogenate were added. To this mixture, 0.2 ml of glutathione followed by 0.1 ml of H_2_O_2_ was also added. The contents were mixed well and incubated at 37 °C for 10 min. A tube containing all the aforesaid reagents except the sample was also incubated as blank. After 10 min, the reaction was stopped by the addition of 0.5 ml of 10% TCA. The tubes were centrifuged and the supernatant was used for the estimation of glutathione by the method of Ellman^27^.

### Statistical analysis

Data are presented as mean ± S.E.M and were analyzed by one-way ANOVA, with post-hoc comparisons by Student Newman-Keuls test using Prism version 4 for Windows (Graph Pad, San Diego, CA). *P* < 0.05 or less was considered as significant.

## Discussion

On the basis of the spectroscopic analyses (Mass, IR, H^1^NMR and ^13^C NMR), the isolated compound from the extract of *M. oliefera* leaves was identified as agnucastoside (7-O-trans-p-coumaroyl-6-O-trans-caffeoyl-8-epiloganic acid). It is an iridoid glycoside, abbreviated as ACC. Although this compound was reported in another plant^14^, for the first time it was isolated from the Moringa leaves. More ever, in this investigation the hitherto unknown potential of ACC has been revealed with regard to the amelioration of ISO-induced cardiac injury and other cardiovascular abnormalities.

Myocardial infarction is studied from different perspectives related to clinical, biochemical, and pathologic characteristics. Electrocardiograph abnormalities are generally considered as diagnostic criteria for the diagnosis of MI. In ISO-induced animals we observed abnormal heart beats and an elevated ST-segment, detected by ECG that might be due to myocardial necrosis caused by ISO. This is supported by a study stating that acute ischemic tissue injury manifests an ST-segment elevation in the region of injured myocardium^33^. However, pretreatment with ACC markedly decreased this ISO-induced tachycardia as well as ST-segment elevation, suggesting the cell membrane protecting effects of ACC, as observed earlier in ISO-induced rats by other workers who used another compound, naringin^34^.

With respect to the alteration of some cardiac markers, after ISO administration, the level of biomarkers, CK-MB and cTnI was increased indicating the occurrence of myocardial necrosis, cellular damage with loss of functional integrity and/or permeability of the cell membrane^35^. This was also supported by an increase in LDH, AST, ALT and heart-to-body weight ratio in ISO-treated rats. Interestingly ACC pretreatment normalized the ISO-induced elevation of serum levels of these diagnostic marker enzymes, suggesting that ACC could maintain membrane integrity, thereby restricting the leakage of these enzymes.

The critical factor in cardiovascular disease is the generation of inflammatory response. Therefore, anti-inflammatory agents play a significant role in attenuation of these responses. Studies have shown that, in myocardial insults, an increase in pro-inflammatory cytokines (e.g., TNF-α and IL-6) and activation of oxidative stress lead to apoptosis and impairment of contractile performance of the heart, sometimes even heart failure^36,37^. Therefore, suppressing TNF-α and oxidative stress cascade is believed to preserve myocardial function. While we observed that ISO induction enhanced both TNF-α and IL-6, which is in accordance with the earlier findings^38^; pretreatment with ACC in ISO-induced rats markedly decreased the levels of both these cytokines suggesting that the cardioprotective effects of ACC may have some relationship with its anti-inflammatory properties.

ISO has been associated with increase in serum lipids, which in turn leads to coronary heart disease^34^. In this study we also observed that in ISO-induced rats there was an increase in the levels of total cholesterol, triglycerides, serum LDL and VLDL with a parallel decrease in HDL cholesterol showing a positive relationship with myocardial infarction. Interestingly, pretreatment with ACC restored these serum lipids near to normal levels, by decreasing most lipids and increasing the HDL cholesterol in ISO-induced rats. An increase in total cholesterol, triglycerides, serum LDL and VLDL could be due to enhanced lipid biosynthesis by cardiac cyclic adenosine monophosphate^37^. Elevated levels of LDL cholesterol and low levels of HDL cholesterol also show a positive correlation with myocardial infarction. In ISO treated animals the triglycerides levels increased significantly which may be due to an increase in its synthesis or could be due to accumulation of acyl CoA and an augmented production of glycerol by increased glycolytic flux, as indicated by previous study^38^. Whatever may be the mechanism for the alterations in different lipids, pretreatment with ACC in this investigation led to a significant improvement in the lipid profile, further supporting its cardio protective role. Evidences also suggest that lipid-lowering property of some therapeutic agents reduces inflammation, which may lower the risk of cardiovascular events^39–41^.

Peroxidation of endogenous lipid might be a major factor involved in the cytotoxic nature of excessive dose of ISO and linked to pathogenic events such as myocardial necrosis, and accumulation of lipid hydroperoxides indicating the damage of the cardiac muscles^42^. In biological system, malondialdehyde, a thiobarbituric acid reactive substance (TBARS) is formed as the end product of plasma membrane associated oxidation of PUFAs and is considered an indicator of lipid peroxidation. Therefore, in this study, an increased level of lipid peroxidation products in ISO-treated rats might have made the cardiac tissues more susceptible to oxidative damage^43^. However, we observed that ACC pretreatment resulted in a significant reduction in TBARS and LOOH in ISO-induced rats showing its anti-lipid peroxidative effect. We further observed a decrease in activities of SOD and CAT in ISO-induced rats which might be due to excessive formation of hydrogen peroxide and superoxide anions^44^. However, our findings clearly demonstrated that ACC pretreatment conserved/increased the activities of these enzymes, suggesting its cardioprotective role. The levels of reduced glutathione and the activities of glutathione peroxidase in the ISO-induced myocardial infarcted rats were significantly decreased, when compared to that in normal control rats. This decrease in GSH level in ISO-treated rat might be due to its increased utilization in protecting–SH group containing proteins from free radicals or due to enhanced protective mechanism to oxidative stress in myocardial injury^45^.

The cardio protective effects of isolated ACC were further supported by direct observations on the myocardial infarction in heart tissues by TTC staining/assay. TTC acts as a proton acceptor for many pyridine nucleotide linked- dehydrogenases along with cytochromes which form an integral part of the inner mitochondrial membrane and make up the electron transport chain^46^. In fact, TTC is known to form a red formazan precipitate with LDH of the viable myocardial tissue, whereas the infarcted myocardium fails to stain with it^46^. We too observed the appearance of patches of pale white color in cardiac tissue slices of ISO treated rats, indicating areas of necrosis due to non-reduction of TTC. Interestingly, prior ACC administration in ISO treated rats protected the heart tissues from infarction, showing reduced infarct size as compared to ISO-induced heart, thus indicating the protection from cardiac necrosis.

During our histopathological investigation, while normal control rats exhibited a clear, intact homogeneous structure of myocardium with no sign of edema and inflammation; administration of ISO caused adverse histopathological changes in the rat myocardium, including cellular necrosis, interstitial edema and inflammatory cell infiltration that are characteristic of MI^47^. However, in rats, pretreated with ACC, although induced by ISO, more or less similar architecture as found in the normal heart (without necrosis and inflammatory cell infiltration, but with intact myofibrils) was observed, showing prevention of necrosis. This protection of myocardial necrosis could have been the result of the antioxidant effects of ACC.

Histopathologial findings were further supported by TEM observations. On one hand the mitochondrial damage resulting from the generation of oxidative stress is a major pathophysiological effect of ISO-induced myocardial ischemia in rats and mitochondrial dysfunction is the key feature of heart diseases. TEM analysis of ISO–induced myocardial tissues revealed fragmentation of myofibrils, swelling of mitochondria with change in shape and size as observed earlier^47^. This mitochondrial swelling could be due to the accumulation of lipid peroxide products as a result of GSH depletion^48^. Interestingly, TEM images of ACC + ISO-treated rats showed regular arrangement of the myofibrils and mitochondria without swelling, as well as regular Z lines. Myofilaments were arranged in a highly ordered fashion between two consecutive Z bands. Thus, ACC prevented swelling of mitochondria, maintaining their function and protected them from the cardiotoxic action of ISO.

The changes in protein expression in the myocardium are critical elements for understanding the molecular mechanisms of cardiac protection. In this study, the proteomics analyses revealed several important proteins, out of which 19 were identified that are related to cardiovascular biomarkers^49^. They belong to the major functional categories such as energy metabolism, heat shock/stress, cytoskeletal function and energy/metabolism. While some proteins were up regulated, others were down regulated following ISO administration and the reverse expressions were observed in the cardiac tissues of ACC pretreated ISO-induced rats.

Two Hsp proteins, α B crystallin and Hsp 27 have been implicated in cardiovascular disease both as a potential biomarker of injury as well as a potential therapeutic target^50^. In the present study, we found that the expressions of two heat shock proteins, Hsp 27 and alpha B crystalline increased in ISO-induced rats, which might be a result of resistance to ISO-induced oxidative stress^50^. However, both the proteins were down regulated by ACC pretreatment and decreased expression was observed in control rats. In ACC pretreated ISO-induced animals a marked decrease in their expression was found indicating that ACC might be inhibiting the synthesis of these proteins and thus protecting the contractile function against the damaging influence of ISO, as suggested by others^51–53^.

Glucose-regulated proteins (GRPs) are also believed to contribute in the maintenance of intracellular Ca^2+^ homeostasis^54^. Following ISO treatment we observed a significant up regulation of GRP 58 kD, a luminal protein of the sarcoendoplasmic reticulum and down regulation of GRP78, a central regulator of endoplasmic reticulum function. Interestingly GRP 58 was down regulated and GRP78 was up regulated in ACC pretreated animals indicating that ACC reduced the oxidative stress in ISO-induced animals and thus it protected the heart from oxidative stress. This finding is somewhat similar to that observed in an earlier report on cortistatin^55^. There appears to be a direct relationship between antioxidant and anti-inflammatory activities as well as in the positive alteration in molecular levels.

When % changes were calculated, it was observed that ACC reduced TNF-α by 27% and IL-6 by 28% in ISO-induced animals, whereas it could increase SOD level up to 77%, Catalase 121% and GSH 130.42% as compared to ISO alone treated animals, suggesting the strong antioxidative potential of ACC, that might be a significant mode of action for the preventive effect of ACC against ISO-induced myocardial injury.

Nine proteins, related to energy/metabolism were identified which are associated with respiratory chain (Rsp-chain), TCA cycle, β-oxidation, glycolysis and phosphocreatine. We found that lactate dehydrogenase B, malate dehydrogenase, aldolase-A, triose-phosphate isomerase, long chain acyl-Co A dehydrogenase and creatine kinase (spot 8, 9, 11, 12, 13 & 17 respectively), were down regulated; whereas, ATP synthtase and triose phosphate isomerase were significantly up regulated in ISO-induced group. Proteomic analysis revealed the low expression of several proteins including, NADH dehydrogenase subunit alpha 10 (NDUFA10, two β-oxidation and TCA cycle related proteins, long-chain acyl-CoA dehydrogenase and malate dehydrogenase (MDH) in ISO treated group, which could be due to decreased oxygen supply and oxidative stress. However, ACC pre-treatment increased the expression of these proteins to the level of control group indicating its ability to scavenge ROS and to improve the provision of energy and the cardiac function^56^.

The proteomic analyses further revealed two proteins, CK and LDH, the expression associated with phosphocreatine and glycolysis, that were down regulated by ISO; but over expressed in the ACC pretreated group, suggesting that the isolated compound promoted glycolysis and phosphorylation preventing energy depletion, thus protected from the myocardial damage, thereby restricting the leakage of these enzymes, as postulated earlier^57^.

The three skeletal proteins, myosin heavy chain 6, myosin light chain 3 and α-actin, associated in the maintenance of cardiac structure and its contractile function, were over expressed in ISO induced animals, but down regulated by the test ACC. The up regulation of these skeletal proteins in ISO-induced animals, as compared to control could be due to the oxidative stress leading to impairment of Ca^2+^ homeostasis causing muscle contractile dysfunction^58^. On the other hand, ACC -induced down regulation of these proteins might have allowed the natural antioxidants to quench the free-radicals, further indicating the cardio protective role of ACC.

Ca^2+^/calmodulin- dependent kinase II (CaMKII), is predominantly expressed in the heart. Over expression of its isoforms δB or δC in ISO-treated rats might have induced hypertrophy as they have specific role in the pathogenesis of cardiac muscles^59^. CaMKIIδC isoform regulates cytosolic Ca^+2^ handling and the δB isoform, localized in the nucleus, regulates gene transcription primarily by increasing the activity of transcription factors of the Mef2 family^60^. In fact, hypertrophic gene expression occurs through phosphorylation of the class II histone deacetylases (HDAC) and subsequent depression of the pro-hypertrophic transcription factor Mef2^61^. As CaMKII inhibition is known to protect against ISO-induced myocyte hypertrophy^62^, its down-regulation could be one of the mechanisms of cardio-protection by ACC. Interestingly, we observed a down-regulation of both δB and δC isoforms of CaMKII. As a representative example, Fig. S4(a) and (b) show a close up view of spot 18 and spot 19 (δB and δC isoforms of CaMKII) in one set of two-dimensional gels and gives statistical data concerning the spot intensity in both groups. In this study, following the pretreatment of ACC, an inhibition of CAMK IIδ isoforms was also noticed. This might have been mediated through the calcineurin (CnA) -dependent signaling pathway because of the fact that in our study all the endogenous antioxidants including SOD were enhanced significantly in ACC pretreated ISO-induced animals. Thus, CaMKII appears to cross-talk with the CnA pathway to affect myocardial hypertrophy. Because CaMKII is activated with enhanced oxidative stress, the protective role of CnA is partially mediated through CaMKII inhibition. In fact, CnA itself is redox regulated and SOD can protect CnA from inactivation^63^. The CaMK II pathway is considered as potential promising target for MI. In fact, in an investigation, marked CaMK II was considered as a clinically important determinant of heart disease and it was suggesed that the inhibition of CaMK II can be a highly selective approach for targeting adverse myocardial remodeling linked to β-AR signaling path way^62^. In the present work, the over-expression of CaMK II was observed in ISO and a down-regulation of δB isoform of CaMKII following ACC administration. These results do suggest that the regulation of CaMK II might be a prominent target for ACC pretreatment against MI.

Due to non-availability of *in vivo* study on ACC, information is lacking on the mechanism or on the pharmacokinetics of ACC. However, some information is there on iridoid glycoside, in which the absorption is believed to be through an active transport mechanism in the small intestine. This may be true in our compound also. As iridoids are primarily metabolized by glucuronide conjugation^64,65^, this possibility cannot be ruled out in our test drug also.

In conclusion, ACC was observed to exert marked cardio protective action through favorable effects on antioxidants, lipid profile, ECG and anti inflammatory factors. Further, the proteomic data indicated that ACC pretreatment to ISO-induced animals preserved mitochondrial function and energy production as well as down-regulated the expression of oxidative stress proteins.

Hence this study recommends the use of isolated compound, ACC for the prevention of myocardial infarction, may be for CVDs in general. However, further studies are required to clearly establish its clinical usefulness in humans.

### Limitations of the study

This study, although reveals the cardio protective effects of a novel compound, ACC it has some limitations. Although the test drug increases the antioxidants and decreases heat shock proteins in ISO-induced rat heart and provide a basis for therapeutic approaches to maintain cardiac function in diseased states, associated with oxidative stress; we didn’t try by coronary artery ligation that is typically performed by an invasive and time consuming approach. In fact, this requires ventilation and chest opening (classical method), often resulting in extensive tissue damage and high mortality. While our experiments clearly demonstrated up regulation and down regulation of some proteins in 2DE, we did not validate it by RTPCR and western blotting to show the relationship between mRNA and protein levels. Of course, mRNA levels may not be necessarily representative of protein levels and, therefore, they cannot provide sufficient data to analyze the mechanism of the progressive cardiac dysfunction after MI. Secondly, we did not measure generation of ROS levels using flow cytometry or EPR. However, in our earlier findings we had established an increase in free radical production in ISO induced animals^66^.

## Electronic supplementary material

Preventive effect of Agnucastoside C against isoproterenol-induced myocardial injury
