# Supplementary material for: Preventive effect of Agnucastoside C against Isoproterenol-induced myocardial injury
Source: Sci Rep. 2017 Nov 23;7:16146. doi: 10.1038/s41598-017-16075-0 (PMC5701045; doi:10.1038/s41598-017-16075-0)
Supplement: Supplementary file 1 — Preventive effect of Agnucastoside C against isoproterenol-induced myocardial injury [file 41598_2017_16075_MOESM1_ESM.pdf]

Supplementary Information

Preventive effect of Agnucastoside *C* against isoproterenol-induced myocardial injury

Sunanda Panda[1*], Anand Kar[1], Sagarika Biswas[2]

[1]School of Life Sciences, Takshashila Campus, Devi Ahilya University, Indore, India.

[2] Department of Genomics & Molecular Medicine, CSIR-Institute of Genomics and Integrative Biology, New Delhi, India.

IR, UV and NMR data of ACC

UV (MeOH) λ_{max} nm (log e): 215 (3.40);

IR: V_{max} (cm^{-1}) 3428, 2908, 1690, 1630, 1510, 1431, 1372, 1228, 1171, 915, 897, 831
HR- ESI-MS: m/z 707 $[M+Na]^+$, $C_{34}H_{36}O_{15}$.

^{1}H NMR data. (400 MHz, DMSO-*d6*, d ppm):5.17(1H d, J=5.5 Hz, H-1), 7.28 (1H, s,H-3), 3.15 (1H, d, J=8.0 Hz,H-5), 1.21 (1H, m) 2.02 (1H, m, H-6), 4.90 (1H, m, H-7) 2.49 (1H, m,H-9), 1.06 (3H, d, J=7.0 Hz,H-10),4.68(d, J=8.0 Hz,H-1'); 3.31 (1H, m,H-2'); 3.40 (1H, d, J=7.0 Hz,H-3'); (3.55 (1H, m, H5'); 4.40 (1H, d, J=, 6.0 Hz,H-6') ; 7.05 (1H, d, J=2.0 Hz,H-2'') ; 6.71 (1H, d, J=8.0 Hz,H-5''); 6.91 (1H, dd, J=8.0, 2.0 Hz,H6"); 6.28 (1H, d, J=16.0 Hz, H-8"); 7.51 (1H, d, J=8.5 Hz, H2''') 6.77 (1H, d, J=8.5 Hz,H-3''');) 6.77 (1H, d, J=8.5 Hz,H-5'''); 6.29 (1H, d, J=16.0 Hz,H 8''')

^{13}C-NMR data.(400 MHz, DMSO-d6) 92.19(C-1); 116.74(C-3); 39.5(C-6); 82.98(C-7); 40.13(C-8); 40.34(C-9),18.37(C-10),74.7(C-2'); 77.1(C-3');70.89(C-4');77.58(C-5'); 63.49(C-6') ; 114.1(C-2'');116.7(C-5'')122.1(C-6''); 146.2(c-7'')115.1(C-8'')166(c-9''); 125(C-1'''); 132(C-2''');116.1(C-3'''),161(C-4'''); 116.2(C-5''')146.2(C-7''');115.2(C-8''');168(C-9''').

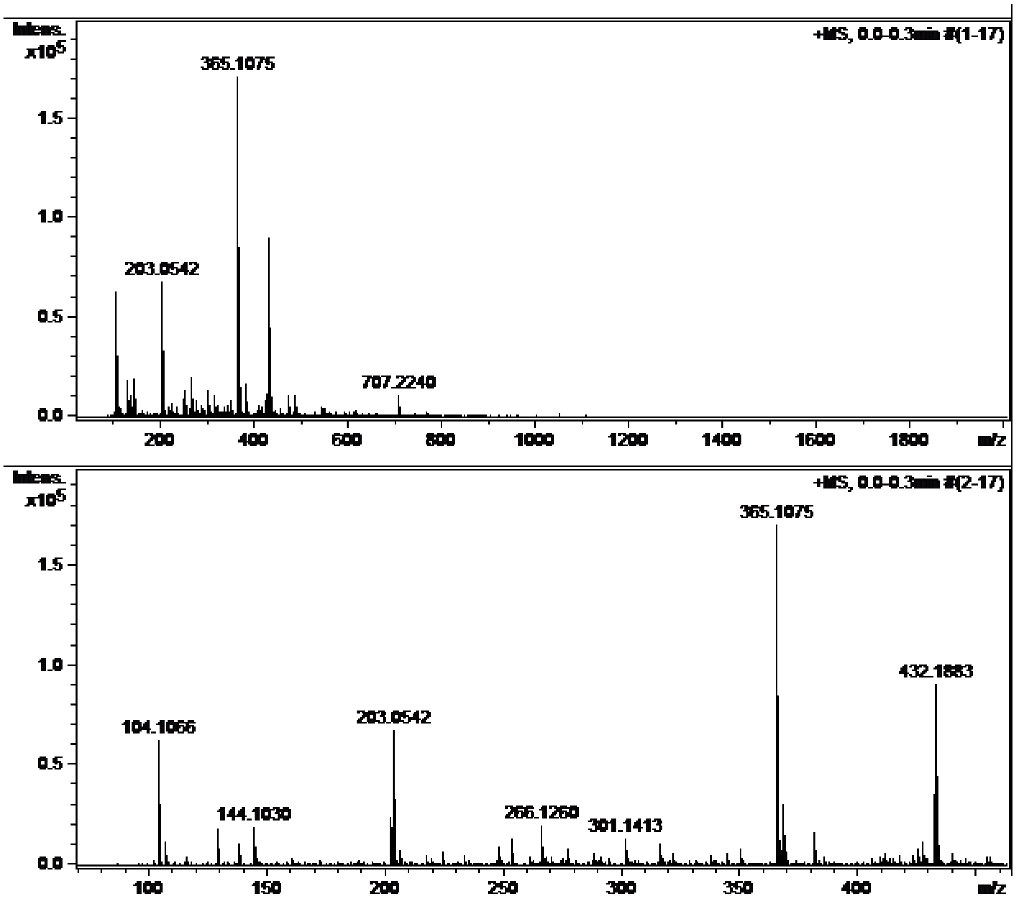

Intens.
x10^5
+MS, 0.0-0.3min #(1-17)
365.1075
203.0542
707.2240
1.5
1.0
0.5
0.0
200
400
600
800
1000
1200
1400
1600
1800
m/z
Intens.
x10^5
+MS, 0.0-0.3min #(2-17)
365.1075
432.1883
104.1066
203.0542
144.1030
266.1260
301.1413
100
150
200
250
300
350
400

Figure S1

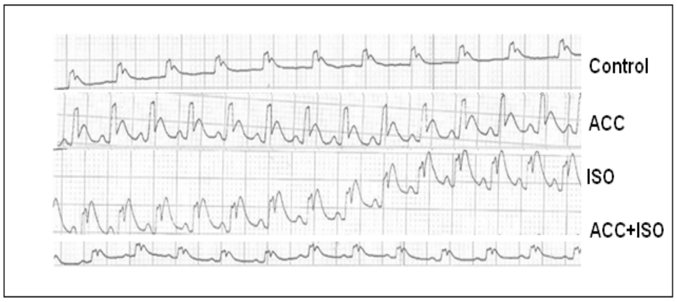

Figure S2(a)

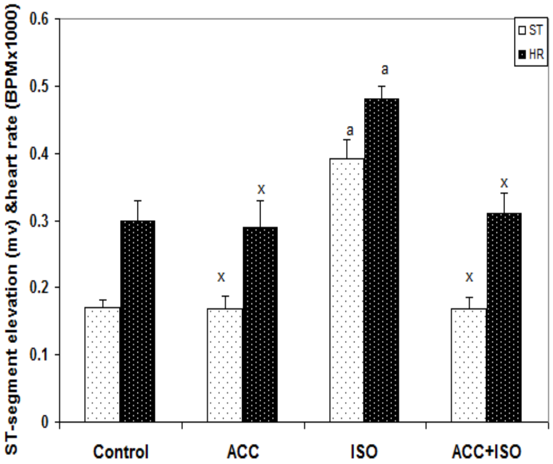

Figure S2 (b)

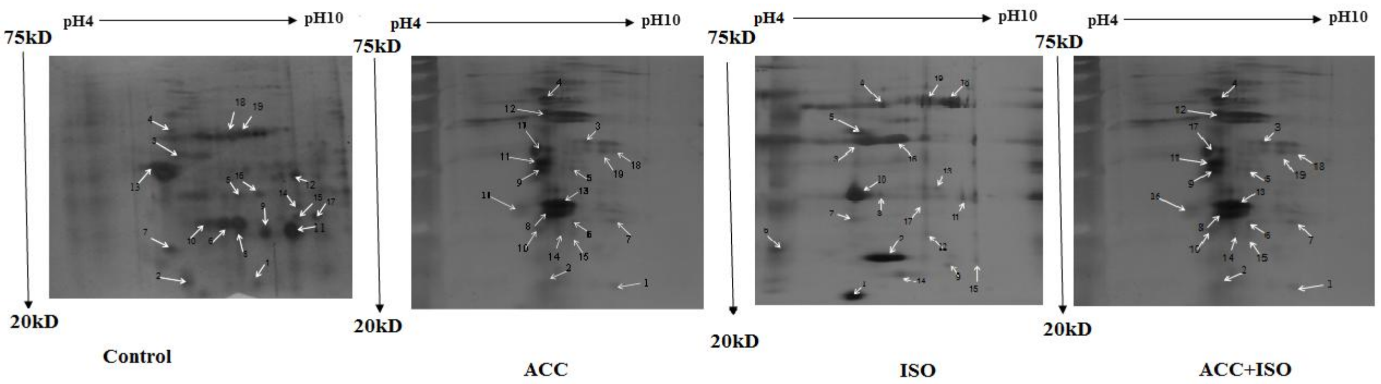

Figure S3

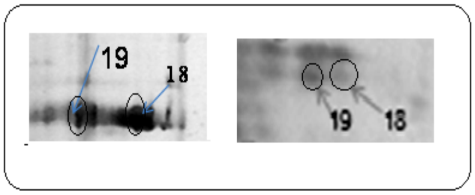

Figure S4 (a)

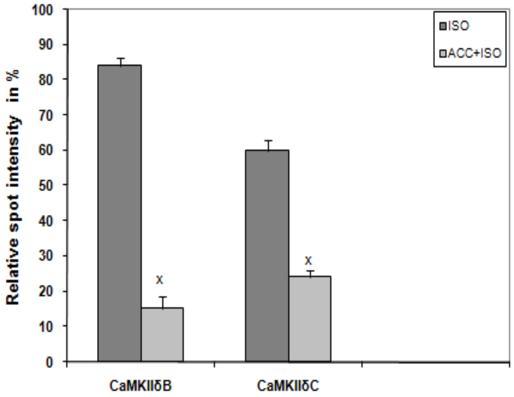

Figure S4(b)

Table S1. Changes in Body weight (B.wt,), Heart weight and Heart weight/Body weight (%) ratio after the administration of ACC.

	Final body weight (g)	Heart weight (g)	Heart weight/B. wt. (%)
Control	183.2 ±10.15	0.610 ±0.041	0.312 ±0.026
ISO	168.4[ns] ±8.67	1.12[a] ±0.088	0.694[x] ±0.029
ACC	194.6 [ns] ±11.10	0.680[x] ±0.061	0.401 ±0.024
ISO+ACC	187.56 ns ±9.93	0.650[x] ±0.051	0.346[x] ±0.030

Data are means ±S.E.M. (n=7). [a]$P<0.001$ as compared to the respective control values. [x]$P<0.001$ as compared to the respective values of the isoproterenol (ISO)-induced animals and ns–non significant (one-way ANOVA, followed by Student Newman-Keuls Post-hoc test).

Table S2 Scoring for histological alterations in myocardium in different groups of rats.

Group(s)	_	+	++	+++
Control	5	0	0	0
ACC	5	0	0	0
ISO	0	0	0	5
ACC+ISO	0	4	1	0

Photomicrographs were used to evaluate the cardiac necrosis in the heart tissue: (-) no changes; (+) mild changes; (++) moderate changes; (+++) marked changes.

Legends to figures.

Figure S1. Mass spectrum of isolated compound, agnucastoside *C* (positive ionization mode).

Figure S2 (a). Representative electrocardiogram (ECG) tracings of control, ACC, ISO and ACC+ ISO treated rats.**(b).** ECG showing changes in ST-segments and in heart beats of control and experimental animals. Values are expressed as mean ±S.E. (n = 7). (One-way ANOVA, followed by Student Newman-Keuls Post-hoc test). ^{a} P<0.001 as compared to the respective control value. ^{x}P<0.001 as compared to the respective value of the ISO- induced animals .

Figure S3. Representative 2D gel images, obtained from heart protein extracts in control, ACC, ISO and ACC pretreated ISO-induced rats.

Figure S4. (a). Selected regions of two-dimensional image showing protein spots 18 and 19, identified as CaMKIIdB and CaMKIIdC. These two spots show higher levels in ISO and lower levels in ACC+ ISO group. **(b).** The figure shows the statistical data of spots 18 and19, displaying a significant decrease in spot intensity in ACC-pretreated ISO-induced rats compared with the ISO-alone group.
